# Use of Prescribed and Non-Prescribed Treatments for Cluster Headache in a Swedish Cohort

**DOI:** 10.3390/brainsci14040348

**Published:** 2024-03-31

**Authors:** Gabriella Smedfors, Felicia Jennysdotter Olofsgård, Anna Steinberg, Elisabet Waldenlind, Caroline Ran, Andrea Carmine Belin

**Affiliations:** 1 Centre for Cluster Headache, Department of Neuroscience, Karolinska Institutet, 171 77 Stockholm, Sweden; gabriella.smedfors@ki.se (G.S.); felicia.jennysdotter.olofsgard@ki.se (F.J.O.); caroline.ran@ki.se (C.R.); 2 Department of Clinical Neuroscience, Karolinska Institutet, 171 77 Stockholm, Sweden; 3 Department of Neurology, Karolinska University Hospital, 171 76 Stockholm, Sweden

**Keywords:** preventive, abortive, side effects, illicit substances, psilocybin

## Abstract

Background: Cluster headache (CH) is a debilitating condition, but current therapies leave CH patients in pain. The extent of this problem in Sweden is unknown. Methods: An anonymized questionnaire was sent to 479 Swedish CH patients to investigate patterns and perceived effects of treatments. Results: Three hundred fourteen answers were analyzed. The population was representative regarding age of onset and sex. Less than half (46%) were satisfied with their abortive treatments, 19% terminated functioning abortive treatments due to side effects. Additionally, 17% of chronic CH patients had not tried the first-line preventive drug verapamil. A small subset had tried illicit substances to treat their CH (0–8% depending on substance). Notably, psilocybin was reported effective as an abortive treatment by 100% (*n* = 8), and with some level of effect as a preventive treatment by 92% (*n* = 12). For verapamil, some level of preventive effect was reported among 68% (*n* = 85). Conclusions: Our descriptive data illustrate that many Swedish CH patients are undertreated, lack functional therapies, and experience side effects. Further studies are warranted to search for new treatment strategies as well as a revision of current treatment guidelines with the aim of reducing patient disease burden to the greatest extent possible.

## 1. Introduction

Cluster headache (CH) patients often suffer from significant personal and economic burden, as well as a high socio-economic load [1,2,3]. Cluster headache is a unilateral primary headache disorder with excruciatingly painful headache attacks lasting between 15 min to three hours. These headache attacks can occur up to eight times a day during active bouts. These severe clinical characteristics along with high personal and societal burden stress the importance of adequate therapy. Despite recent research advancement, absence of complete understanding of the pathogenesis complicates the development of novel therapies. Prevailing consensus, however, does find this neurovascular headache to anatomically involve the posterior hypothalamus, brainstem, and midbrain, and it manifests through activation of cranofacial parasympathetic fibers as well as trigeminal nerve fibers [4]. One prominent symptom of CH is its rhythmicity of attacks reported by a majority of patients and possibly linked to the circadian rhythm [5]. 

Currently, CH is pharmacologically treated in part by aborting individual attacks (high-flow oxygen; oral, nasal or injectable triptans), in part by transitional treatment (corticosteroids such as prednisolone), and in part by preventive oral treatment (e.g., verapamil and lithium) or injectable treatment (e.g., Galcazenumab) aimed to reduce attack frequency and/or induce/prolong the remission period [6,7]. Unfortunately, neither line of treatment is fully effective: 22–53% are still in pain after abortive treatments; transitional treatment has only temporary effects; and 21% of CH patients are not helped by the first line of preventive treatment verapamil [7,8]. Adding to the complexity are the side effects associated with the treatments [6]. Taken together, some CH patients remain debilitated by their disease and seek novel ways to control it, including non-approved medications and illicit substances, primarily in the form of cannabis and psychedelics.

Cannabis use has been reported more elevated in the CH population [9,10] but with variable effects [11]. According to a report by the Swedish Health Agency from 2020, cannabis use is generally lower in Sweden than many other European countries. Cannabis use is most prevalent in the age group 16–34 with an estimated 8% of men and 5% of women that age using it (https://www.folkhalsomyndigheten.se/livsvillkor-levnadsvanor/andts/andts-anvandning-och-ohalsa/anvandning/narkotikaanvandningen-och-utvecklingen/cannabis-och-folkhalsa/ (accessed on 12 March 2024)). Though the Swedish Health Agency reports a more positive attitude toward cannabis use as compared to other narcotics in the general Swedish population, the majority still believe it should not be legal. Epidemiological data of psychedelic substance use in Sweden are limited, although it is predicted to be low. One of the main reasons for the low number of users is the strict laws governing cannabis and psychedelic use in Sweden. Cannabis and psychedelic drug use, possession, and distribution are completely outlawed in Sweden with a few exceptions for medical use and can lead to a substantial fine or up to three years in prison.

The so called “classical psychedelics” such as psilocybin and lysergic acid diethylamide (LSD), active via the serotonin 2A (5-HT2A) receptor, have been reported to have therapeutic effects in CH [12]. The knowledge of psychedelics as medicine is rapidly increasing and includes sparse dosing, a favorable safety profile, and lack of addictive properties [13,14,15,16]. Retrospective data on the therapeutic effect of psychedelic substances against CH describe abortion of acute attacks, termination of cluster periods, remission induction, and prolonged remission [9,17,18,19]. Psilocybin has been shown to be effective in clinical trials as well. Two clinical trials investigating preventive effects of psilocybin on CH have been published. A Danish study reports a 30% reduced attack frequency in patients suffering from chronic CH. Also, a negative correlation of changes in hypothalamic–diencephalic functional connectivity with relative reduction in attack frequency was found [20]. The second study by Schindler et al. recruited both episodic (ECH) and chronic (CCH) CH patients. Though not significant, they found a 31.3% weekly reduced attack frequency, with a large effect size in chronic patients (d = 1.25) [21]. Interestingly, no correlation between the hallucinogenic state and symptom relief was detected. A third clinical trial investigating the matter is ongoing (NCT03781128). Additionally, psilocybin has shown some effectiveness for other primary headache disorders; one pilot study finding a decrease in attack intensity and frequency in migraine patients for two weeks after a low dose of psilocybin [22].

As optimized treatment regimens are needed to minimize CH disease burden, increased knowledge of treatment usage, effects, and side effects of prescribed and non-prescribed treatments including illicit substances is crucial, especially when reports on therapeutic potential from illicit substances in the form of psychedelic substances are accumulating. Since this has not previously been carried out, the aim of this study was to investigate the perceived effects of these treatments and drugs among Swedish CH patients.

## 2. Materials and Methods

A 57-item questionnaire was sent to 479 CH patients via Karolinska Institutet’s “KI Survey” platform during June–October 2021 (Supplementary Materials). Answers were obtained from 318 respondents (66.4% response rate), Supplementary Figure S1. The questionnaire and informed consent were sent to the study participants via email. The study was approved by the Swedish Ethical Review Authority in Stockholm, Sweden (diary number 2014/656-31/4). Respondents were either already a part of the existing CH biobank at Karolinska Institutet or recruited through an advertisement in *HuvudJournalen*, the magazine of the Swedish headache patient organization, Huvudvärksförbundet. Respondents from the cohort (92.4%) have a validated CH diagnosis by a neurologist (co-authors A.S. or E.W.) according to the International Classification of Headache Disorders (ICHD-III beta) criteria [23]. The remaining 7.6% patients were self-reported CH. Four individuals were excluded from the analysis because of incompletion of the survey (defined as less than 20%). On average, participants answered 93% of the questions. For a few of the questions, some of the participants answered “Yes” to multiple alternatives. In this case we chose to keep the alternative, which stated the highest effect (full effect was kept as opposed to, moderate, partial, or none). Participants also had the possibility to provide free-text answers, which were included in the analysis. If the free-text answer for response was incomplete, the answer was reported as usage only. Participants were asked about Erenumab, Galcanezumab, and Fremanezumab separately; however, the number of participants who had tried CGRP monoclonal antibodies was low and therefore the data were combined.

### Statistical Analysis

Data were downloaded from the KI survey and processed in R/RStudio [24,25]. Satisfaction of abortive treatment was compared between men and women and CH subtypes using Pearson’s chi-square test and in relation to number of drugs tried using ANOVA. Categorical data were described as percentages from (a) all respondents and (b) those who tried the substances/treatments. 

## 3. Results

The respondent cohort consisted of 38.5% (*n* = 121) women and 61.5% (*n* = 193) men, out of which 73.6% (*n* = 231) suffered from ECH and 26.1% (*n* = 82) from CCH. One individual had an unclear subtype. The mean age of disease debut was 29.6 years. We had information from 157 of the 164 individuals who did not respond to or did not complete the survey. This group consisted of 40.8% (*n* = 64) women and 59.2% (*n* = 93) men; 82.2% (*n* = 129) were ECH, 14.7% (*n* = 23) CCH and five with unclear subtype. The mean age of disease debut was 34.7 years. 

### 3.1. Abortive Treatments

In total, 46.5% of study participants were satisfied with their abortive treatment, 33.8% were somewhat satisfied, 16.9% were not satisfied, and 2.9% did not answer. When looking at the genders individually, 51.3% of men were satisfied with their abortive treatment, while only 38.8% of women were satisfied. This difference was not statistically significant (*p* = 0.4). No statistical difference was found between the subtypes, although 25.6% of CCH patients were not satisfied with their abortive treatment as compared to 13.9% of ECH patients (Table S1).

**Figure 1:**
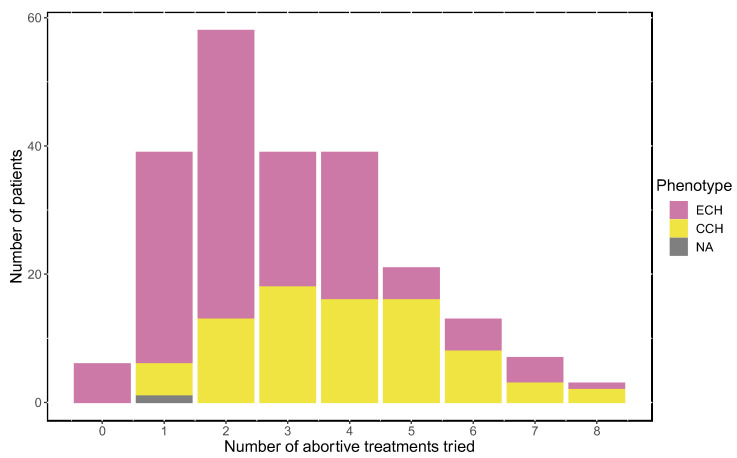
The number of prescribed abortive treatments tried. ECH = episodic cluster headache (*n* = 225); CCH = chronic cluster headache (*n* = 82); NA = subtype not available (*n* = 1). Individuals who did not reply to any of the questions regarding acute treatment were excluded from this analysis (*n* = 6).

Most study participants had tried more than one abortive treatment for CH attacks (Figure 1). More specifically, 52.8% of ECH patients and 76.8% of CCH patients had tried three treatments or more. We also saw that 97.4% of the study participants had tried an abortive treatment. The most frequently reported number of medications tried were two for ECH (25.1%) and three for CCH (22.0%). Statistical analysis showed no correlation between number of treatments tried and satisfaction with the abortive treatment (*p* = 0.58).

**Figure 2:**
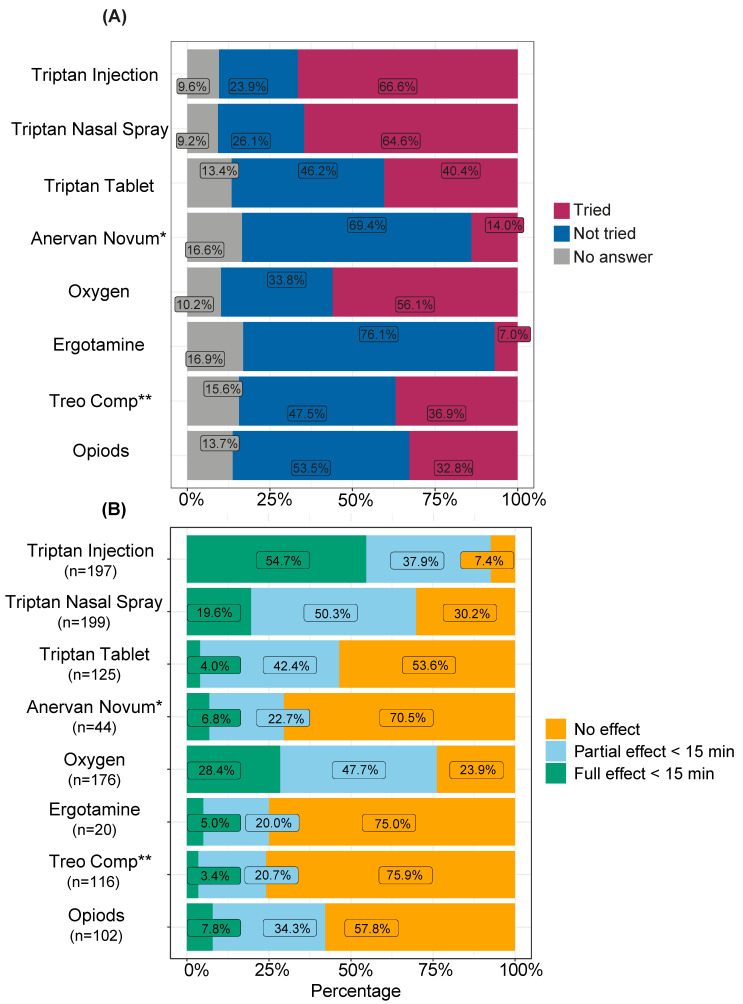
Usage (**A**) and effect (**B**) of prescribed abortive treatments in cluster headache. (**A**) Usage of drugs to abort a cluster headache attack, proportions are reported as % of the respondent cohort. (**B**) Effect of drugs used to abort a cluster headache attack with percentages based on the study participants who had actively tried the substance in (**A**). * Anervan Novum contains Ergotamine, Chlorcyclizine and Caffeine. ** Treo Comp contains acetylic acid and codein.

The most frequently reported effective abortive treatment was triptan injections, which had full effect within 15 min in 54.7% and partial effect within 15 min in 37.9% among the 66.6% who had tried it (Figure 2A,B and Table S2). The second most reported was oxygen treatment, which had a full effect in 28.4% and partial effect within 15 min in 47.7%. 

Side effects from abortive treatments were reported by 40.8%, and 18.5% reported that they had chosen to discontinue an otherwise effective treatment due to side effects. Out of the 32 free-text answers, triptans were mentioned in 54.4% of them as the major source of side effects among abortive treatments. The side effects mentioned were chest pain, nausea, fatigue, anxiety, abnormal heart rate, dizziness, numbness in arms, muscle pain, increased headache afterward, and nosebleed. When asked about termination due to side effects of otherwise functioning treatments, triptans were also the most frequently reported substance (30 of the 58 free-text answers).

#### Non-Prescribed Abortive Treatments

**Figure 3:**
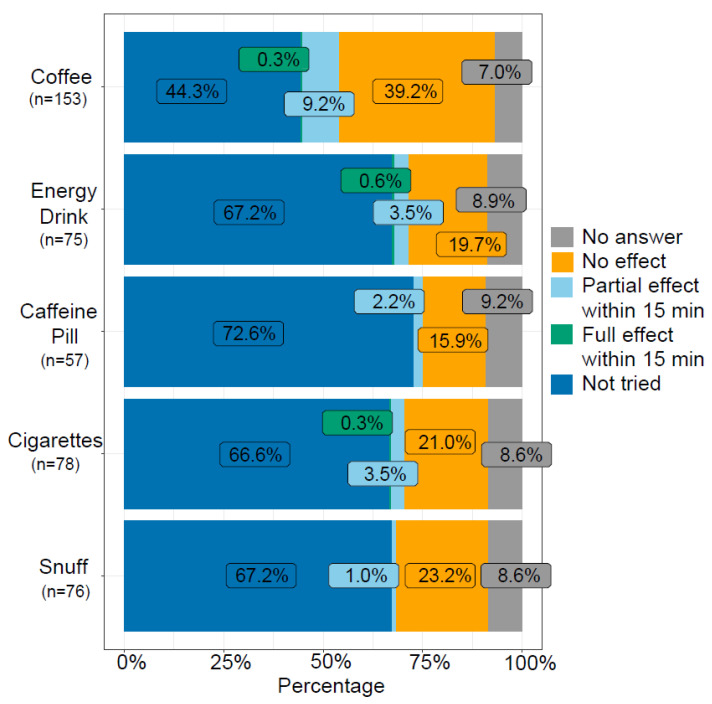
Effect of non-prescribed substances used to abort a cluster headache attack. Proportions are reported as % of the respondent cohort.

A substantial number of patients had not tried coffee or nicotine as treatment alternatives and among those who had tried, a vast majority answered that they did not have any effect (Figure 3). However, 19.6% (*n* = 30) reported that coffee had a pain-alleviating effect within 15 min. While one study participant reported full abortive effect within 15 min from cigarettes, and 3.5% (*n* = 11) reported partially alleviating effect within 15 min from cigarettes, two participants wrote in the free-text answer that cigarettes/nicotine could provoke attacks. 

Few study participants reported use of the illicit substances psilocybin, LSD, N,N-Dimetyltryptamin (DMT) and cannabis, 0–8.0% depending on substance (Table 1). No respondent reported use of 5-methoxy-N,N-dimethyltryptamin (5-MeO-DMT), N,N-di allyl-5-methoxy tryptamine (5-MeO-DALT), lysergic acid amide (LSA) seeds, or 2-Bromo-LSD (BOL-148). Only one study participant reported use of DMT and this with full abortive effect. Psilocybin had an attack abortive effect in all study participants who reported use of it (*n* = 8). LSD had full abortive effect within 15 min in the four study participants who had tried it. Cannabis gave rise to pain relief in 56.0% of the users (*n* = 25).

### 3.2. Preventive and Transitional Treatment

In the respondent cohort, 27.4% of the study participants reported that they were satisfied with their preventive treatment, 32.2% were somewhat satisfied, and 26.8% were not satisfied. No response was reported in 13.7%. No differences between genders or subtypes were identified.

**Figure 4:**
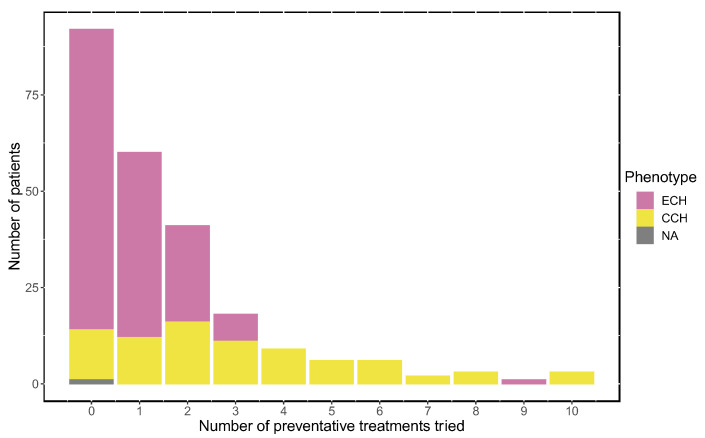
The number of prescribed preventive treatments tried. ECH = episodic cluster headache (*n* = 229); CCH = chronic cluster headache (*n* = 82); NA = subtype not available. Individuals who did not reply to any of the questions regarding acute treatment were excluded from this analysis (*n* = 2). Preventative treatments did not include nerve interventions.

Not having tried preventive treatments (excluding nerve interventions) was the most common answer among ECH patients (39.8%). A fourth (26.0%) of ECH patients had tried one preventive treatment and 17.7% had tried two. Among CCH patients, the groups who had tried 0, 1, 2, or 3 preventive treatments were more similar in size (respective % = 17.1%; 14.6%; 19.5%; 13.4%). Almost exclusively, CCH patients had tried four or more preventive treatments (Figure 4). Further, statistical analysis showed a correlation between having tried many prophylactic treatments and being less satisfied with the treatments (*p* = 0.013).

**Figure 5:**
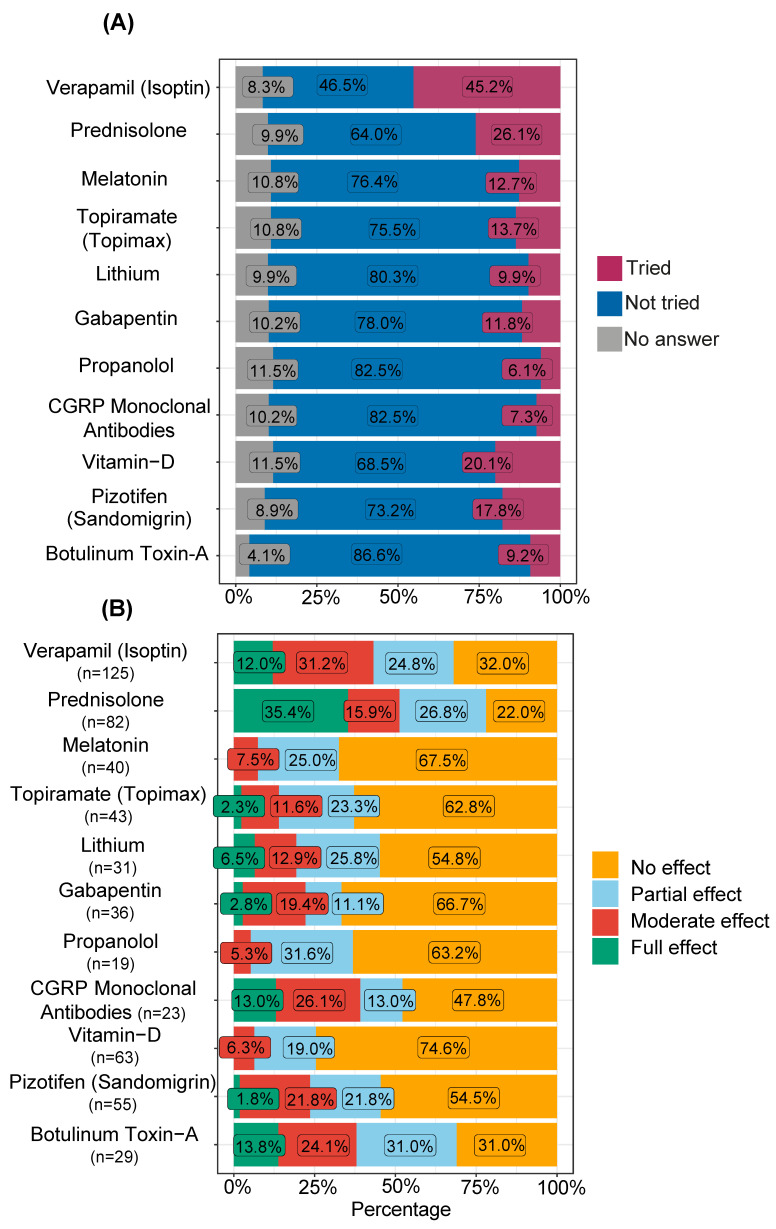
Self-reported usage and effect of preventive and transitional treatments for cluster headache. (**A**) Usage of drugs to prevent cluster headache (CH) attacks/bouts, proportions are reported as % of the respondent cohort. (**B**) Effect of drugs to prevent CH attacks/bouts with percentages based on the study participants who had actively tried the substance noted in (**A**).

Of those who had tried the first-line preventive treatment verapamil, 10.5% reported full effect and 28.1% reported no effect (Figure 5 and Table S3). Prednisolone was reported with full effect by 35.4% and 22.0% reported no effect. Botulinum toxin-A injections were fully effective among 13.8% and had some level of effect (full, moderate, or partial) in 69.0% of those who had tried. Calcitonin gene-related peptide (CGRP) antibodies treatment was used by relatively few patients (7.3%) but this was the fourth most efficient treatment in preventing CH symptoms. With the exclusion of verapamil, prednisolone, botulinum toxin-A, and CGRP antibodies, all treatments were more often reported with no effect than with all levels of effect taken together (full, moderate, and partial). 

Side effects from preventive treatments were reported by 29%. In the 93 free-text answers, verapamil was most frequently mentioned (*n* = 21), followed by topiramate (*n* = 13), prednisolone (*n* = 10), lithium (*n* = 6), gabapentin (*n* = 5), and amitriptyline (*n* = 4). The remaining answers were unspecific. The side effects for verapamil were constipation, fatigue, abnormal pulse/heart activity, nausea, weight gain, anxiety, and water retention. Topiramate was mentioned as causing fatigue, hair loss, cognitive distortions, abnormal heart activity, depression, anxiety, enhanced perception of sound and colors, speech difficulties, weight loss, and loose stool. Prednisolone was reported to cause stress, insomnia, weight gain, acne, redness, water retention, and high blood sugar. Amitriptyline caused weight gain, low blood pressure, and negative impact on mood. Discontinuation of otherwise functioning preventive treatment(s) due to side effects was reported by 9.9%. The discontinued drugs were written as free text. Verapamil was mentioned x7, topiramate x5, lithium x2, and “all of them” by three study participants. 

Nerve interventions were tried by a subset of the cohort with Cefaly/TENS (transcutaneous electrical nerve stimulation) as the most common intervention tried by 11.5%, followed by botulinum-toxin A (9.2%). For Cefaly/TENS, no one reported full effect and 61.1% reported no effect (Table 2). 

#### Non-Prescribed Preventive Regimens

Few patients reported use of illicit substances for preventive purposes, 0–6.4% depending on the substance (Table 1). Of those who tried psilocybin, 58.3% reported full preventive effect and 91.7% experienced some level of effect (full, moderate, or partial). For LSD, 75.0% reported full preventive effect and 87.5% had some level of effect. Two study participants reported use of DMT and 5-MeO-DMT, respectively, with full preventive effect for one of the participants and none or partial effect for the other participant. Cannabis gave rise to full preventive effect in 25.0% and to partial preventive effect in 55.0% of the study participants, while 45.0% reported no effect (Table 3).

### 3.3. Free-Text Answers

#### Illicit Substances

One percent of the study participants and twenty-five percent of individuals who take psilocybin as a preventative treatment report that they use some kind of *busting method.* This is when one consumes a planned, often stepwise, regimen of a lower dose of psychedelic substance (often psilocybin) to keep the attacks at bay [26]. Quotes from the free-text answers on busting methods are available in Supplementary Materials.

## 4. Discussion

The descriptive data from our online self-reported survey illustrate how Swedish CH patients often suffer from sub-optimal treatment both in the sense of lacking efficacy and from side effects. Swedish CH patients often try a series of medications during the course of their disease, with a majority of patients having tried three or more abortive treatments. Additionally, a number of preventive treatments need to be tried, especially for CCH patients where almost half of them have tried three or more alternatives. Still, despite these efforts less than half are satisfied with their acute treatments and almost one in three stands without satisfactory preventive treatment. At the same time, 40.0% of ECH patients and 17.1% of CCH patients have not tried any preventive treatments at all. This survey also reveals how almost one in five patients discontinue otherwise functioning abortive medications due to side effects.

The treatment response from triptan injections is higher in our cohort (92.6%) than in a previous study, where 74% experienced pain relief within 15 min [27]. Interestingly, more than 90% of those who tried triptan injections acquired either full or partial effect, but almost one in four had not tried this alternative. Not having tried triptan injections could be due to contraindications or experience of side effects from other forms of triptans, e.g., tablets. Prescribing tablets before nasal or injection triptans can, in addition, delay optimal abortive treatment. Given their effect, triptan injections or nasal spray, should always be considered when prescribing abortive treatments. In line with previous data, triptans in the form of nasal spray are reported as less efficacious than injections [28]. The data also reveal how about a third of the patients have tried opioids as an abortive, even though these substances are not standard treatment in CH. A considerable percentage of individuals have never tried oxygen to treat their CH attacks (33.8%). It is in line with other studies looking at medication use in CH, although it is important to note that oxygen is seen as one of the best tolerated and efficient treatments for CH [29]. Finding the correct flow rate and oxygen mask can be crucial to having an effect from high-flow oxygen treatment and therefore could partially explain the 23.9% of individuals who had no effect from oxygen. Anecdotally, patients have previously reported that caffeine and nicotine can help to abort attacks. This has a rationale as both substances are involved in pain modulation through their action on adenosine and nicotinic acetylcholine receptors [30,31]. In our survey, caffeine in the form of coffee had the highest perceived effect (*n* = 30) and nicotine in the form of cigarettes was reported effective by 11 responders. 

For all preventive medications included in this study apart from verapamil, a majority of patients had not tried them, and full effect was only reported by a few users. Whether or not this is due to a misconception of the potential of preventive drugs to induce remission rather than decreasing the attack frequency or length is not known. Nevertheless, it is noteworthy that 46.5% of the cohort had not tried the first-line preventive drug verapamil (Figure 5A). This is troublesome given the reduction in disease burden verapamil often offers CH patients. The effect of verapamil in our cohort was lower than in a previous study, with 12.0% describing full preventive effect, compared to 27% [32]. When also including moderate and partial effects from verapamil, 68.0% of those who had tried it experienced an effect. This is still less than in two previous studies in which ~80% were helped [33,34]. In this report, we found that CGRP antibodies treatment was reported as the fourth most efficient preventative treatment for CH. Results from clinical trials with CGRP antibodies have been ambiguous, and only one treatment is currently approved by the U.S. Food and Drug Administration, whereas the European Medicines Agency has not approved this for ECH patients [35]. Our data show a reasonably good treatment effect with 52.2% experiencing full, moderate, or partial effect. Considering the safety profile of these treatments, our results suggest that the recommendation for these drugs should be revisited. 

Treatments involving nerve interventions such as botulinum toxin-A and nerve stimulating interventions were also reported with limited effects, but it is worth noting that these therapies are more likely to be prescribed to chronic patients who are generally more refractory to treatment. However, botulinum toxin-A injections had the highest number of users with an effect and the lowest number of users without effect (Figure 5B and Table 2). This should motivate botulinum toxin-A injections before more invasive interventions, as the effect of botulinum toxin-A injections comes with relatively few side effects [36]. 

Only a small subset of the respondents had tried illicit substances against their CH, 0–8.0%, depending on substance (Table 1 and Table 3). The most-used illicit substance among the responders was cannabis. However, approximately half reported no positive effect from cannabis, neither as an acute nor as a preventive treatment. Our data are consistent with a previous report on cannabis use in French CH patients, where over 50% of the study participants reported uncertain or variable effects of cannabis on CH [11]. Still, cannabis use has been reported more elevated in the CH population [9,10], and therefore it cannot be excluded that the endocannabinoid system is involved in the pathophysiology. Also in our cohort, cannabis use was more common than what is reported by Sweden’s public health agency for the general population in Sweden, 8.0% vs. 3.3% in Swedish people aged 16–64 (https://www.folkhalsomyndigheten.se/livsvillkor-levnadsvanor/andts/andts-anvandning-och-ohalsa/anvandning/narkotikaanvandningen-och-utvecklingen/cannabis-och-folkhalsa/ (accessed on 12 March 2024)). Cannabis has often been described as a potential agent of pain relief, and cannabinoid-based drugs are suggested as treatment for many disorders manifesting with pain, e.g., multiple sclerosis and cancer, but there is little evidence of cannabis as treatment for headache, (for review see reference [37]). Notably, pain management with cannabis of conditions with a persistent pain profile, differs substantially from the restricted CH pain. Also, placebo due to disproportionate positive media attention is a confounder in pain management with cannabinoids [38]. 

Use of psychedelic substances was sparsely reported in our cohort compared to a previous Dutch study [9]. LSD was used at similarly low rates in Dutch CH patients, whereas psilocybin was more commonly reported in the Netherlands as compared to Sweden, possibly due to more permissive drug policies in the Netherlands. Importantly, in both our and the Dutch study, these compounds are reported to have a good acute and preventive effect on CH, comparable to or superior to prescribed treatments. Noteworthy, the effects from psychedelics are acquired after limited drug dosing (i.e., 1–3 doses), in contrast to traditional treatments, including cannabis, which are taken daily to maintain disease suppression. The reason for this unusual effect seen in psychedelics is not known.

Psychedelic substances were used by 0–3.8% of our cohort but were reported with full or partial effect as an abortive treatment by all who had tried. As preventive treatment, psilocybin was reported to have at least partial effect in 91.7%, and 58.3% reported full preventive effect. For those who had tried LSD, 87.5% reported at least partial preventive effect, and 75.0% reported full preventive effect. These elevated numbers can be compared to full effect for 54.7% of triptan injection users and 12.0% for verapamil. A placebo effect may account for some of the effects reported because use of psychedelic substances is frequently mentioned as highly effective, e.g., in patient forums and in media. Why these effects are reported by users of psilocybin and LSD is not known, but notably these substances do in-part signal via the same serotonergic receptor as triptans, 5-hydroxytryptamine receptor 1B (5-HT1B) [7,39], which could explain the abortive effects.

Limitations to our study concern that data are self-reported and retrospective, which may introduce recall bias, and may affect the recollection of tried substances, effects, and side effects. Also, comorbidities, other medications, disease duration, and severity were not taken into account when considering treatment choice, success, or failure. In addition, self-selection bias might occur, e.g., patients with a higher disease burden may be more motivated to participate or, on the contrary, have less energy to participate in a research study. Non-responders to the survey tended to have a higher age of disease debut and were less likely to be chronic CH patients. This supports the idea that patients with a higher disease burden are more likely to answer the survey and this should be considered when interpreting the results. The answers may also be affected by the participant being in or out of bout. We also lack information about doses and treatment length, including the formulation of verapamil, which can be both slow and fast acting and therefore affect the effectiveness. To support recollection, free-text answers were allowed for most questions to allow reporting of drugs not listed in the question. Self-reported data also come with advantages: reporting of illicit substance use can be considered a stigma and/or risk, thus there is a risk of underreporting. In an anonymized web-based questionnaire like our survey, there is a higher probability that patients report usage of these drugs. Also, an additional disclaimer was added in the survey, that the data would be analyzed anonymously. An additional strength of our study is that the cohort was very well characterized, and almost all the study participants had been diagnosed by a neurologist. Furthermore, the Swedish healthcare system subsidizes the prescribed treatments discussed in this paper, and therefore underreporting due to poor income or lack of insurance should be close to zero.

## 5. Conclusions

Optimized treatment regimens are needed to minimize CH burden. Gaps of effective treatments are apparent, and troubling side effects are common in this Swedish cohort. In addition, surprisingly few patients are using available preventive treatments. A small subset of the study participants report use of other strategies such as non-prescribed substances, nerve manipulation, and illicit substances as an attempt to manage CH. Of these, psilocybin was reported as most effective, both to abort attacks and for prevention. This study shows that CH patients in Sweden are undertreated, possibly because of low efficacy of existing treatments and/or insufficient care.

## Figures and Tables

**Table 1:** Effect of illicit substances as acute abortive treatment of cluster headache attacks.

Substance	Proportion of Cohort Who Tried (n)	Full Relieving Effect within 15 min (n) *	Partial Relieving Effect within 15 min (n) *	No Effect (n) *
Psilocybin	2.5% (8)	37.5% (3)	62.5% (5)	0% (0)
LSD	1.3% (4)	100% (4)	0% (0)	0% (0)
DMT	0.3% (1)	100% (1)	0% (0)	0% (0)
Cannabis	8.0% (25)	24% (6)	32% (8)	44% (11)

LSD: lysergic acid diethylamide, DMT: N,N-Dimetyltryptamin. * Based on study participants who had actively tried the substance.

**Table 2:** Effect of nerve stimulation interventions on cluster headache attacks.

Method	Proportion of Cohort Who Tried (n)	Full Effect (n) *	Moderate Effect (n) *	Partial Effect (n) *	No Effect (n) *
Cefaly/TENS	11.5% (36)	0.0% (0)	8.3% (3)	30.6% (11)	61.1% (22)
Vagus-nerve stimulation	5.1% (16)	0.0% (0)	12.5% (2)	37.5% (6)	50.0% (8)
SPG	4.1% (13)	23.1% (3)	7.7 (1)	15.4% (2)	53.8% (7)
DBS	1.9% (6)	16.7% (1)	33.3% (2)	16.7% (1)	33.3% (2)

TENS: transcutaneous electrical nerve stimulation, SPG: sphenopalatine ganglion block, DBS: deep brain stimulation. * Based on study participants who had actively tried the substance.

**Table 3:** Effect of illicit substances as preventive treatment for cluster headache attacks.

Substance	Proportion Who Tried (n)	Full Effect (n) *	Moderate Effect (n) *	Partial Effect (n) *	No Effect (n) *
Psilocybin	3.8% (12)	58.3% (7)	25.0% (3)	8.3% (1)	8.3% (1)
LSD	2.5% (8)	75% (6)	12.5% (1)	0% (0)	12.5% (1)
DMT	0.6% (2)	50% (1)	0% (0)	0% (0)	50% (1)
5-MeO-DMT	0.6% (2)	50% (1)	0% (0)	50% (1)	0% (0)
Cannabis	6.4% (20)	25% (5)	10% (2)	20% (4)	45% (9)

LSD: lysergic acid diethylamide, DMT: N,N-Dimetyltryptamin, 5-MeO-DMT = 5-methoxy-N,N-dimethyltryptamin. * Based on study participants who had actively tried the substance.

## Data Availability

The data presented in this study are available on request from the corresponding author. The data are not publicly available due to privacy restrictions.

## Supplementary Materials

The following supporting information can be downloaded at: https://www.mdpi.com/article/10.3390/brainsci14040348/s1, Figure S1: Flow diagram showing inclusion and exclusion of cluster headache patients for participation in questionnaire study; Table S1: Subtype satisfaction of abortive treatment; Table S2: Usage of prescribed abortive treatment for cluster headache attacks; Table S3: Usage of preventive or transitional treatment for cluster headache; Questionnaire and Free-text answers.
