# Supplementary material for: Use of Prescribed and Non-Prescribed Treatments for Cluster Headache in a Swedish Cohort
Source: Brain Sci. 2024 Mar 31;14(4):348. doi: 10.3390/brainsci14040348 (PMC11048603; doi:10.3390/brainsci14040348)
Supplement: Supplementary file 1 [file brainsci-14-00348-s001.zip › brainsci-2894836-supplementary.pdf]

Supplementary material for:

Use of prescribed and non-prescribed treatments for cluster headache in a Swedish cohort

Flow diagram showing inclusion and exclusion of cluster headache patients for participation in questionnaire study.

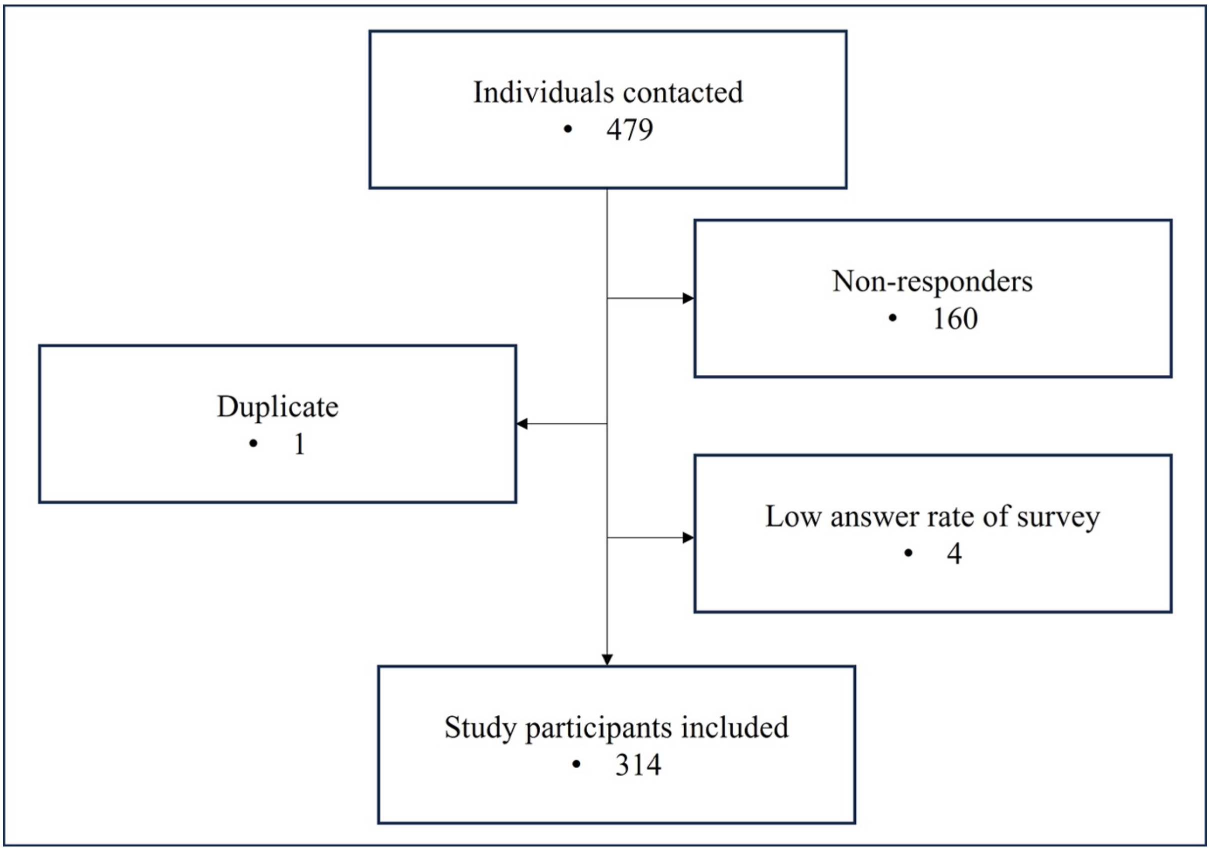

Figure S1. 479 individuals were recruited for inclusion in our cluster headache biobank. 175 were excluded due to; had not replied at the specific time point of data collection (n=160), duplicate (n=1), low answer rate of survey (n=4). The study included 314 participants. A G44.0 diagnosis was not confirmed in 24 and in total 290 study participants validated with a G44.0 diagnosis participated in this questionnaire study.

Table S1: Subtype satisfaction of abortive treatment

**Subtype**	**Satisfied**	**Somewhat satisfied**	**Not satisfied**	**No answer**
ECH	49.4% (114)	34.2% (79)	13.9% (32)	2.6% (6)
CCH	37.8% (31)	32.9% (27)	25.6% (21)	3.7% (3)

ECH = episodic cluster headache, CCH = chronic cluster headache

Table S2: Usage of prescribed abortive treatment for cluster headache attacks

**Substance**	**Tried Substance (n)**	**Not Tried Substance (n)**	**Not Answered (n)**
Triptan Injections	66.6% (209)	23.9% (75)	9.6% (30)
Triptan Nasal Spray	64.6% (203)	26.1% (82)	9.2% (29)
Triptan Pill	40.4% (127)	46.2% (145)	13.4% (42)
Anervan Novum	14.0% (44)	69.4% (218)	16.6% (52)
Oxygen	56.1% (176)	33.8% (106)	10.2% (32)
Ergotamine	7.0% (22)	76.1% (239)	16.9% (53)
Acetylic Acid + Codein	36.9% (116)	47.5% (149)	15.6% (49)
Opiods	32.8% (103)	53.5% (168)	13.7% (43)

Table S3: Usage of preventive or transitional treatment for cluster headache

**Substance**	**Tried Substance (n)**	**Not Tried Substance (n)**	**Not Answered (n)**
Verapamil (Isoptin)	45.2% (142)	46.5% (146)	8.3% (26)
Prednisolone	26.1% (82)	64.0% (201)	9.9% (31)
Melatonin	12.7% (40)	76.4% (240)	10.8% (34)
Topiramate (Topimax)	13.7% (43)	75.5% (237)	10.8% (34)
Lithium	9.9% (31)	80.3% (252)	9.9% (31)
Gabapentin	11.8% (37)	78.0% (245)	10.2% (32)
Propanolol	6.4% (20)	82.2% (258)	11.5% (36)
Aimovig (Erenumab)	4.8% (15)	84.7% (266)	10.5% (33)
Emgality (Galcanezumab)	2.9% (9)	86.3% (271)	10.8% (34)
Ajovy (Fremanezumab)	3.5% (11)	85.7% (269)	10.8% (34)
Vitamin-D	20.1% (63)	68.5% (215)	11.5% (36)
Pizotifen (Sandomigrin)	17.8% (56)	73.2% (230)	8.9% (28)
Botulinum Toxin-A	9.2% (29)	86.6% (272)	4.1% (13)

Questionnaire

Welcome to a survey for patients with cluster headache

The point of this survey is to attain information which only you as a patient living with cluster headache can give. This information is crucial in order to be able to create the most optimal treatment plan for individuals with cluster headache.

This survey is about treatment, both the treatment prescribed by doctors and other alternative methods.

Thank you for participating in our study by answering the following questions!

This is how you fill in/answer the paper version of the survey
Below you can see how you should mark an answer, and how you can change an answer if you regret your preliminary choice

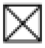 Correctly marked answer

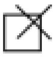 Incorrectly marked answer, the cross should be in the middle of the square 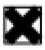 Incorrectly marked answer, the cross is too bold

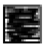 Regret preliminary choice, the answer will not be counted as marked

I consent to the usage- and integrity conditions of the study "Identifying, modeling, and characterizing genetic factors pertaining to cluster headaches" according to Attachment 1 v.04 (attached to the study mail invite).

☐ Yes

☐ No

Part 1: Treatment of cluster headache

1. How old are you?

[ ]

2. Gender

☐ Woman

☐ Man

☐ Do not want to say

3. How tall are you (cm)?

[ ]

4. How much do you weigh (kg)?

[ ]

5. In which country are you born?

[ ]

6. Do you have cluster headache?

☐ Yes

☐ No, not anymore, but I had it earlier in life

7. What type of cluster headache do you have?

☐ Episodic

☐ Chronic

Definition Episodic cluster headache:
Cluster headache attacks coming in recurring bouts. Cluster headache bouts last from seven days to one year. There should be three months or longer remission periods in between bouts.

Definition Chronic cluster headache:
Recurring cluster headache attacks during more than one year without remission or with shorter remission periods which last less than three months.

Optional comment:

8. At which age did you get your first cluster headache attack?

9. If you do not get cluster headache attacks anymore, when was the last time you had an attack?

Optional comment:

10. Are/were you satisfied with your acute medication for cluster headache attacks (to abort an attack)?

☐ Yes

☐ No

☐ Rather

Optional comment:

11. Which of the following do you use or have used as acute treatment for cluster headache? With what effect?
e.g. Triptans:
Injeection: Imigran, Sumatriptan SUN
Nasal spray: Imigran, Zomig
Tablet: Maxalt, Sumatriptan, Zomig

	No effect	Partial effect within 15 minutes	Full effect within 15 minutes	Have not tried it
Triptan injection	☐	☐	☐	☐
Triptan nasal spray	☐	☐	☐	☐
Triptan tablet	☐	☐	☐	☐
Anervan Novum	☐	☐	☐	☐
Relpax	☐	☐	☐	☐
Oxygen	☐	☐	☐	☐
Ergotamine	☐	☐	☐	☐
Treo Comp	☐	☐	☐	☐
Opioids (e.g. Citodon or OxyNorm)	☐	☐	☐	☐

Any other substance which is not stated in the list? Effect?

12. Have you experienced physical and / or psychological side effects from any of your acute treatment? E.g. chest pain, weight gain, headache, or anxiety.

☐ Yes

☐ No

If yes – substance(s) and side effect(s):

13. Have any of these side effects been so troublesome that you have decided to stop using the substance even though it worked well against cluster headache attacks?

☐ Yes

☐ No

If yes – substance(s) and side effect(s):

14. At most, how many times per day do you use triptans (listed above)?

☐ 0
☐ 1
☐ 2
☐ 2-4
☐ 4-6
☐ More than 6

Do you experience that something limits the number of doses of triptans per day you take? E.g. doctor's prescription or side effects.

15. Do you use other substances which are not pharmaceutical drugs in the acute treatment of cluster headache?

	No effect	Partial abortive effect within 15 minutes	Full abortive effect within 15 minutes	Have not tried it
Coffee	☐	☐	☐	☐
Energy drinks	☐	☐	☐	☐
Caffeine tablets	☐	☐	☐	☐
Cigarettes	☐	☐	☐	☐
Snuff	☐	☐	☐	☐

Any other substance which is not stated in the list? Effect?

16. Are you satisfied with your preventive medication for cluster headache?

☐ Yes
☐ No
☐ Rather

Optional comment:

17. Which of the following substances have you used as preventive medication for treating cluster headache?

	No effect	Partial effect	Moderate effect	Full effect	Have not tried it
Verapamil/Isoptin	☐	☐	☐	☐	☐
Prednisolone	☐	☐	☐	☐	☐
Melatonin	☐	☐	☐	☐	☐
Topiramate/Topimax	☐	☐	☐	☐	☐
Lithium	☐	☐	☐	☐	☐
Gabapentin	☐	☐	☐	☐	☐
Propranolol	☐	☐	☐	☐	☐
Aimovig (Erenumab)	☐	☐	☐	☐	☐
Emgality (Galcanezumab)	☐	☐	☐	☐	☐
Ajovy (Fremanezumab)	☐	☐	☐	☐	☐
Vitamin-D	☐	☐	☐	☐	☐
Sandomigrin	☐	☐	☐	☐	☐

Any other substance which is not stated in the list? Effect?

18. Have you experience any physical and/or psychological side effects from any of your preventive medication? E.g. chest pain, weight gain, constipation, fatigue, or anxiety.

☐ Yes

☐ No

If yes – substance(s) and side effect(s):

19. Have any of these side effects been so troublesome that you have decided to stop using the substance even though it worked well against cluster headache attacks?

☐ Yes

☐ No

If yes – substance(s) and side effect(s):

20. Have you tested any apparatus for nerve stimulation or undergone any surgical interventions to alleviate the symptoms of cluster headache?

	No effect	Partial effect	Moderate effect	Full effect	Have not tested
Botox injections	☐	☐	☐	☐	☐
Cefaly / TENS	☐	☐	☐	☐	☐
Stimulation of the vagusnerve	☐	☐	☐	☐	☐
Deep brain stimulation (DBS)	☐	☐	☐	☐	☐
Stimulation of the sphenopalatine ganglion (SPG).	☐	☐	☐	☐	☐

Any other method which is not stated in the list? Effect?

Besides the above mentioned substances patients have increasingly testified that alternative substances can help against cluster headache. To further investigate what the optimal treatment for cluster headache could be, it is important to collect information regarding these substances and their effects. Your answers will never be shared or spread and will only be used strictly for research.
All analysis and reports will be done based on group comparisons of anonymous data which means that no identification of unique individuals is possible. The answers can thus never be held against you legally.

21. Have you used any of the substances mentioned below as acute treatment to abort a cluster headache attack?

	No effect	Partial abortive effect within 15 minutes	Full abortive effect within 15 minutes	Have not tried it
Psilocybin ("magic mushroom")	☐	☐	☐	☐
LSD	☐	☐	☐	☐
DMT	☐	☐	☐	☐
5-MeO-DMT	☐	☐	☐	☐
5-MeO-DALT	☐	☐	☐	☐
LSA seed	☐	☐	☐	☐
BOL-148	☐	☐	☐	☐
Cannabis	☐	☐	☐	☐

Any other substance which is not stated in the list? Effect?

22. Have you used any of these substances to decrease the number of your cluster headache attacks (as a form of preventive treatment) or in order to extend a remission period between attack periods?

	No effect	Partial effect	Moderate effect	Full effect	Have not tried
Psilocybin ("magic mushroom")	☐	☐	☐	☐	☐
LSD	☐	☐	☐	☐	☐
DMT	☐	☐	☐	☐	☐
5-MeO-DMT	☐	☐	☐	☐	☐
5-MeO-DALT	☐	☐	☐	☐	☐
LSA seed	☐	☐	☐	☐	☐
BOL-148	☐	☐	☐	☐	☐
Cannabis	☐	☐	☐	☐	☐

Any other substance which is not stated in the list? Effect?

23. Have any of the above mentioned substances decreased your ailments, e.g. fewer or less painful cluster headache attacks?

☐ Yes

☐ No

☐ Do not know

Describe how:

24. Have any of the above mentioned substances terminated an active attack period/bout?

☐ Yes

☐ No

☐ Do not know

Which/what substance(s)?

25. A) Have any of the mentioned substances kept you in remission during an extended period of time?

☐ Yes

☐ No

☐ Do not know

Which/what substance(s)?

B) How long did these substances allow you to remain in remission (what was your longest remission period after taking one or more of these substances)?

26. "Busting method" Some cluster headache patients use substances according to a certain schedule or a certain method, ex 1 per week for 3 consecutive weeks, twice a year. Do you do that?

☐ Yes

☐ No

27. If you use a specific method: Describe how you use the substance. What dose and how often?

28. If you do *not* use a specific method: What dose and how often do you use the substance?

29. Have any of these side effects been so troublesome that you have decided to stop using the substance even though it worked well against cluster headache attacks?

☐ Yes

☐ No

If yes – substance(s) and side effect(s):

30. Do you experience that your cluster headache (diagnosis?) has contributed to:

	No effect	Decreased	Increased	Not relevant
Anxiety	☐	☐	☐	☐
Depression	☐	☐	☐	☐
Social isolation	☐	☐	☐	☐
Suicidal thoughts	☐	☐	☐	☐
Self harm	☐	☐	☐	☐

Optional comment:

31. Have any of the substances mentioned above had an effect on:

	No effect	Decreased	Increased	Not relevant
Anxiety	☐	☐	☐	☐
Depression	☐	☐	☐	☐
Social isolation	☐	☐	☐	☐
Suicidal thoughts	☐	☐	☐	☐
Self harm	☐	☐	☐	☐

Which substances?

IMPORTANT Psychological ailments are firstly handled by your health care clinic (vårdcentral). More information regarding this can be found via the 1177 Healthcare guide (Vårdguiden). If your mental health gets dramatically worse contact your closest psychiatric emergency room, call the suicide hotline at the phone number 90101 or call 112.

The questions below are pertaining to the substances mentioned in the previous page. If you haven't tried any of these substances, you can skip to the next page.
Psilocybin ("magic mushroom"), LSD, DMT, 5-MeO-DMT, 5-MeO-DALT, LSA, BOL-148, Cannabis, Another substance which you specified

32. How did you initially come in contact with these substances?

At what age?

33. How do you feel about taking substances which are considered illegal?

34. What does the substances cost?

35. Is it difficult to get a hold of the substance?

☐ Yes

☐ No

How do you get access to the substance?

36. Do you have any comments regarding the questions about treatment?

Example of quotes from the free text answers for question 28 on busting methods;

If you use a specific method: Describe how you use the substance. What dose and how often?:

- "If I start to go into a relapse, I take psilocybin about once a week. Every three months or so if I have no problems."
- "Yes, I usually try to get mushrooms in me every three months, a little more often during the winter as it is my high season for cluster headache ."
- "When I have felt that it starts to look like a relapse, I have taken a half or a quarter "blotter" with LSD" (a blotter is a small piece of absorbent paper containing the drug)."
- "Micro dose before recurring attack periods."
- "One blotter (LSD) once a month. Or 5 g of mushrooms once a month."
- "Microdosing every third day. About 0.3 grams (psilocybin)."
- "I've gone years without getting hold of LSD or mushrooms and those periods in life have been heavy or heavier than usual."
- "I take psilocybin relatively regularly as a preventive measure, especially if I feel like I'm on the way to a relapse but experience the "side effects", i.e. the psychedelic state is too stressful at times, so it depends a little on my mental state."
